# Kinetic, Spectral, and Structural Characterization of a Heme-Containing Peroxidase From the Skin of *Cucurbita maxima*

**DOI:** 10.1155/bri/7629890

**Published:** 2025-10-05

**Authors:** Alexis Jackson, Isabelle Thompson, Ellen Cochrane, Ashton Ware, Rachel Byrum, Lydia Buxa, Carson Farmer, Hector Medina, Gregory M. Raner

**Affiliations:** ^1^Department of Biology and Chemistry, Liberty University, Lynchburg, Virginia, USA; ^2^School of Engineering, Liberty University, Lynchburg, Virginia, USA

**Keywords:** biodegradation, catalysis, peroxidase, PFAS

## Abstract

A heme-containing peroxidase was isolated and characterized from the skin of *Cucurbita maxima* (PKS). Large-scale purification of the enzyme was performed, yielding a stable and active preparation suitable for detailed biochemical analysis. The PKS's properties were investigated, including pH sensitivity, temperature stability, and the influence of ionic strength on its activity and its spectroscopic properties. Kinetic parameters were determined using fluorophenol substrates and compared to those of the extensively studied horseradish peroxidase (HRP), highlighting similarities and unique features. The primary structure determination of the purified PKS was conducted using protease digestion and subsequent MS fragment analysis. Coupled with genomic data from PKS, a corresponding protein sequence was determined, which was then used to generate a 3-dimensional structure for the PKS enzyme using computational approaches. In addition, a spectrophotometric analysis of the purified PKS was conducted, enabling the determination of molar extinction coefficients for the holoenzyme in its presumed 5-coordinate high-spin ferric state (as isolated) and its 6-coordinate high-spin ferric state upon complexation with fluoride ions. The dissociation constant for fluoride binding exhibited significant pH sensitivity, consistent with observations reported for HRP. Additionally, the PKS enzyme showed distinct spectral binding features when complexed with trifluoroacetic acid, whereas no such binding was observed with HRP. These findings provide insights into the biochemical and structural characteristics of this low-cost plant peroxidase. Furthermore, our results provide relevant information for advancing the potential uses of these peroxidases in biotechnological applications such as environmental remediation of perfluorinated carboxylic acids.

## 1. Introduction

Peroxidase enzymes have proven to be valuable tools in biotechnology with applications in areas such as biosensor development, photocatalytic reactions, and bioremediation solutions, just to name a few. For decades, the peroxidase isolated from horseradish root (HRP) has served as the paradigm for understanding peroxidase chemistry and development of novel applications for these versatile catalysts [1–5]. The utility of this particular enzyme lies in its abundance in the horseradish root, its high catalytic activity, and, more recently, the availability of a recombinant system for overexpression and isolation. Interest in the identification and characterization of peroxidases from other plant sources, however, has been increasing in recent years [6–13]. For example, peroxidases from avocado [6], zucchini [7], water crest [8], garlic [9], corn [10], plums [11], ginger [12], asparagus [13], and cucumber [14, 15], just to name a few, have been isolated and characterized over the past several decades, with applications ranging from the biobleaching of industrial dyes to degradation of chlorinated phenolics in contaminated soil. In each of the cases listed, however, the peroxidase concentration in these sources is low relative to the horseradish root, making the isolation and purification from these plant sources more costly or with lower yield.

Recently, a peroxidase enzyme isolated from the skin of pumpkins (*Cucurbita maxima*) was shown to effectively degrade a series of mono- and polyfluorinated phenols with activities comparable or superior to those of HRP [16]. Furthermore, the yield of peroxidase per gram of tissue from pumpkin skin (PKS) exceeded that of horseradish root considerably. The bioaccumulation of fluorinated aromatics in the environment has made the search for effective biocatalysts for their efficient degradation a high priority [17–20], and the high peroxidase content in this abundant plant source makes the pumpkin an excellent candidate for further development in this and potentially other applications. However, very little is currently known about the PKS, and even its primary structure had not been elucidated up to this current study. Furthermore, in the reactions with fluorinated aromatics, detailed kinetic analyses were not carried out, so the more precise kinetic parameters associated with the reactions remain to be elucidated. In the current study, a large-scale purification of the PKS enzyme was carried out, and the isolated enzyme was characterized regarding pH sensitivity, temperature stability, and influence of ionic strength. Detailed kinetic parameters were also established for the PKS enzyme and in its degradation of 4-fluorophenol (4FP), and in each case, these properties were compared with those of the well-characterized peroxidase, HRP. In addition, the purified enzyme was analyzed by protease digestion and mass spectrometry (MS) sequencing and, combined with the genomic information available for *C. maxima*, a protein sequence has been determined. From this sequence, a 3-dimensional model of the PKS was generated computationally and compared with HRP. Finally, a spectrophotometric characterization of the pure enzyme was performed, and molar extinction coefficients for the holoenzyme were determined for the ferric form as isolated, and the hexacoordinate high-spin ferric complex formed in the presence of fluoride ions. The dissociation constant for fluoride binding to the PKS was found to be highly sensitive to pH, as also demonstrated previously for HRP [21]. Like HRP, PKS was found to bind acetate in the active site, favoring an apparent 5c high-spin complex; however, PKS was also shown to bind the perfluorinated derivative, trifluoroacetic acid, in the same manner, whereas HRP did not accommodate this ligand under the same conditions. The results of these binding studies are discussed in the context of prior studies describing the oxidation of carboxylic acids such as acetic acid by the horseradish peroxidase enzyme [22], and the newly determined structure of the enzyme.

## 2. Materials and Methods

### 2.1. Purification of PKS

Approximately 450 g of the peel from two large pumpkins (*C. maxima*) was removed and processed by passage through a manual wheatgrass press along with 50 mL of phosphate buffer (20 mM at pH 7.0). This produced about 200 mL of a crude liquid sample, which was centrifuged for 15 min at 8000 × *g* at 4°C. To the cleared supernatant, ammonium sulfate solid was added to give a 40% saturated solution on ice. The sample was stirred for 10 min at 0°C and centrifuged for 10 min at 8000 × *g* at 4°C. The pellet was discarded, and this supernatant was then loaded onto a 25-mL phenylsepharose column equilibrated with equilibration buffer (potassium phosphate buffer, 20 mM at pH 7.0 that was 40% saturated with ammonium sulfate). A deep red band appeared at the top of the column as the crude sample loaded, and following a 20-mL wash with the equilibration buffer, the PKS was eluted using 20-mM potassium phosphate buffer, pH 7.0. As the red band began to elute, 5-mL fractions were collected, and those fractions with the highest activities were pooled to give a final volume of approximately 22 mL. Aliquots of this purified enzyme were subjected to SDS for analysis of purity and molecular weight.

### 2.2. Electrophoresis

Electrophoresis was carried out using a Biorad gel system and precast 10% SDS-PAGE gels (Mini-PROTEAN TGX) purchased from Biorad. Biorad Precision Plus Protein Kaleidoscope prestained MW markers were run alongside the samples, which were treated with 2× Laemmli SDS-sample buffer (Bioworld) heated to 95°C for 5 min and loaded onto the gel. The electrophoresis was carried out for 30 min at a constant voltage of 200 V and stained overnight using Coomassie blue stain. After destaining (40% methanol and 10% acetic acid), gel was imaged using a ChemiDoc MP imaging system, and the band corresponding to the PKS enzyme was cut out using a razor blade for subsequent sequencing analysis. The gel slice was covered in 200 μL of H_2_O in a microcentrifuge tube and mailed on ice to the Duke Proteomics and Metabolomics facility (Durham, NC) for proteolytic MS analysis.

### 2.3. Spectrophotometric Properties of the Purified PKS

A Thermo Scientific Genesys 150 spectrophotometer was used for all measurements. The scanning mode was set to collect at a rate of 5 nm/sec with 1-nm resolution. The absorption spectra for a 16-μM solution of both PKS and HRP in 50-mM phosphate buffer (pH 7.0) were acquired. Titrations of PKS with KF were carried out via addition of 1-μL aliquots of a 1.0-M KF stock solution to a 2.0-mL solution containing 18-μM PKS in 100-mM phosphate buffer at either pH 4.8 or pH 6.0. Trifluoroacetic acid and acetate binding to PKS and HRP were also monitored in a similar fashion using a stock solution of 6.5-M TFA (or 8.5 M acetic acid), with 1-μL aliquots added to a 1.0-mL solution of either PKS (11.0 μM) or HRP (10 μM) in 300-mM phosphate buffer at the designated pH. A simple 1 : 1 ligand binding model was used to determine *K*_*d*_ for fluoride binding (equation (1)); here, the fraction of the enzyme bound was determined spectrophotometrically using Δ*A*/Δ*A*_max_, where Δ*A* is the change in absorbance at 403 nm upon addition of ligand, and Δ*A*_max_ is the absorbance change under saturating ligand conditions. To calculate *K*_*d*_, we used a double reciprocal plot from equation (1), shown in equation (2), where −1/*K*_*d*_ is represented by the *X*-intercept of the linear plot.

(1) $$\begin{array}{c} \Delta A=\frac{\Delta A_{\max}\;\left[L\right]}{K_{d}}+\left[L\right], \end{array}$$

(2) $$\begin{array}{c} \frac{1}{\Delta A}=\frac{K_{d}}{\Delta A_{\max}}\times \frac{1}{\Delta A_{\max}}+\frac{1}{\Delta A_{\max}}. \end{array}$$

### 2.4. Heme Content Determination

Extinction coefficients were determined for the PKS enzyme by measuring the precise concentration of heme in enzyme samples, based on the assumption that the purified PKS contains a single heme cofactor. Heme content for the purified enzyme was measured using an HPLC method in which the enzyme is first treated with a 50 : 50 mix of acetonitrile and water, each containing 0.1% TFA (Solvent A), and incubated 5 min prior to injection on a C8 HPLC column (50 × 4.6 mm) and subjected to chromatography in Solvent A at a flow rate of 1.0 mL/min with detection at 403 nm. The HPLC system used was an Agilent Integrity 1260–automated HPLC system with a quaternary mixing system and in-line degassing coupled to diode array absorption detection module. A sharp heme peak was observed at 2.5 min under these conditions. A standard curve was prepared using a commercial sample of horseradish peroxidase where the concentration of the standard solution was determined spectrophotometrically using an *ε*_403_ = 1.02 × 10^5^ M^−1^ [23]. The standard curve generated by this method was shown to be linear over the range of 0.05 μM–100 μM (100 μM was the highest concentration used, so an upper limit on the linear range could not be established). The same HPLC method was used to monitor heme modification in the reactions with acetate buffer.

### 2.5. Sequencing Analysis

Pure PKS samples for sequencing were produced by carrying out SDS-PAGE on the purified enzyme and subsequently cutting out the band from the gel. Characterization was carried out at the Duke University's Proteomics and Metabolomics Center. Using the NCBI genomic database, MS analysis of the tryptic digest from the pure enzyme produced fragmentation patterns that were compared to all predicted enzymes in the genome for *C. maxima*, and a peroxidase sequence was identified from the database that matched the predicted pattern and MW for the predominant enzyme in the purified sample.

### 2.6. Kinetic Assays

To assess activity of the peroxidase enzymes, two different assays were employed. A continuous assay involving the spectrophotometric monitoring of guaiacol oxidation was used, where the activity for each sample was determined in units (1/2 × μmoles tetraguaiacol produced per min). An ε_490_ value for tetraguaiacol of 26.6 mM^−1^ was used. In addition, a discontinuous assay was employed involving the oxidation of 4FP into benzoquinone [16]. To determine optimum conditions for the PKS, pH (4.5–8.5), ionic strength (25–510 mM), and substrate concentration (2.0–40 mM) of the reaction mixtures varied; all other parameters for the kinetic analysis were similar to those reported in [16]. A phosphate buffer was used in all cases, and the ionic strength of the phosphate buffer was calculated using equation (3), where *c*_*i*_ was the concentration of each ion in the buffer and *z*_*i*_ is the charge on that ion.

(3) $$\begin{array}{c} I=\frac{1}{2}\overset{n}{\underset{i=0}{\sum }}c_{i}z_{i}^{2}. \end{array}$$

The 3 ions used for this calculation were K^+^, HPO_4_^−2^, and H_2_PO_4_^−1^, where each individual species was calculated using the Henderson–Hasselbalch equation:

(4) $$\begin{array}{c} \text{pH}=\text{pKa}+\log\frac{K_{2}{\text{HPO}}_{4}}{KH_{2}{\text{PO}}_{4}}. \end{array}$$

Partitioning studies were carried out to determine the ratio of quinone produced to substrate degraded. This was done by measuring the decrease in the peak area for 4FP at 275 nm and converting it to a percent decrease in concentration, then comparing this to the increase in benzoquinone concentration observed over the same time period using the standard curve method.

To determine the temperature stability of the enzymes, an assay was developed in which enzyme samples in 50-mM phosphate buffer, pH 6.5, were preincubated at temperatures between 50 and 70°C for up to 60 min. An aliquot of the pretreated enzyme was removed at the prescribed time and added to a reaction mixture containing 5.0 mM H_2_O_2_ and 5.0 mM 4-FP in 50-mM phosphate buffer (pH 6.5) to initiate the reaction. After 2 min, the reaction was quenched and analyzed by HPLC, as described previously [16].

## 3. Results and Discussion

### 3.1. Purification of PKS

**Figure 1:**
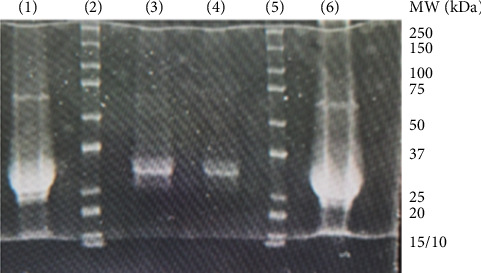
SDS-PAGE results showing the purified PKS using the scheme described. Lanes (1) and (6) correspond to undiluted PKS enzyme. Lanes (2) and (5) are the MW markers and lanes (3) and (4) are the PKS at a 1:10 dilution.

From approximately 450 g of PKS, 365 mg of PKS enzyme were purified using a simple 2-step purification procedure involving ammonium sulfate precipitation and hydrophobic chromatography on a phenylsepharose column.  Figure 1 shows the SDS-PAGE results for the purification scheme previously described, demonstrating the effectiveness of the procedure. The outer lanes represent the undiluted PKS enzyme following purification, while the inner two lanes are a 1 : 10 diluted sample of this purified enzyme. Separating the two samples on each side are the molecular weight markers. Based on the gel migration in SDS-PAGE, an approximate molecular weight of 29,000 Da was determined. The abundance and ease of purification of PKS are two of the properties making it an excellent candidate for exploration in a number of different peroxidase applications, as suggested in our previous work [16].

### 3.2. Primary Structure

**Figure 2:**
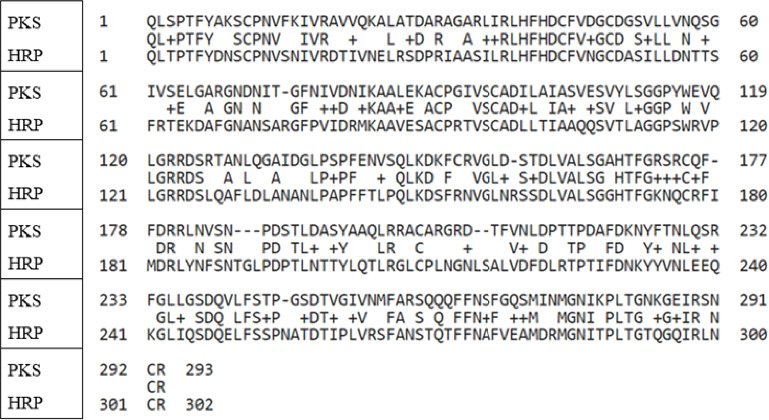
NCBI BLAST analysis comparison of primary structure of HRP and that of the PKS enzyme determined in the current study.

Based on the MS analysis of tryptic digests of the purified PKS, along with the genomic information available for *C. maxima*, the primary structure of the enzyme was determined and showed 52% sequence identity with HRP based on an NCBI BLAST analysis. An overlay of the PKS and HRP sequences is shown in Figure 2. Conserved regions surrounding the heme-binding pocket can be seen, as expected for the Class III peroxidases. For example, the active site residues F142, F179, H170, F41, H42, R38, and P139 from HRP [24] are all conserved in PKS. There are, however, several differences in the active site and region surrounding the substrate access channel that are of note. For example, phenylalanines at Positions 68 and 221 in HRP are replaced with arginine and leucine, respectively, in PKS. Phe 68 is positioned above the δ-meso face of the heme cofactor in HRP, which is the site of approach for peroxidase substrates, while Phe 221 is on the proximal face of the heme below the γ-meso carbon on the heme. Prior studies involving the PKS enzyme suggested slight alterations in the reactivity of fluorinated phenolic compounds with the pumpkin peroxidase when compared to HRP, which could potentially be explained by these observed differences in structure [16]. The experimentally determined molecular weight of the PKS of 29,000 Da, as determined by SDS-PAGE, is slightly lower than the predicted value based on the sequence analysis, which was 32,000 kDa, and a predicted pI of 8.9 was obtained for this enzyme, compared to 8.8 for HRP.

**Figure 3:**
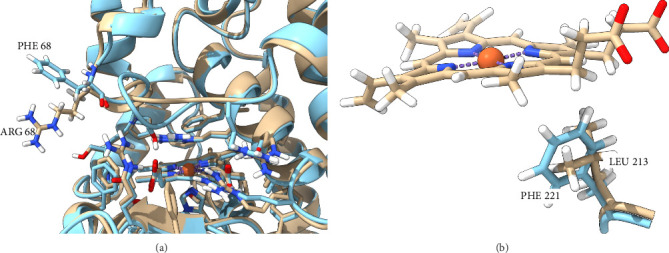
Structure of the PKS enzyme determined using AlphaFold 3 molecular modeling software. The modeled sequence is aligned with the crystal structure for the HRP-C protein. (a) The “gating” Phe at position 68 in HRP and the corresponding Arg in PKS are labeled. (b) The locations of the Phe at position in 221 in HRP and Leu in 213 in PKS.

The primary structure determination also opens the door for more detailed structural characterization via in silico methods. For example, Figure 3 is a protein structure generated using Alphafold 3 software based on primary structure information. For comparison, the HRP structure is also given. Of particular note is the residue mentioned previously at Position 68 in HRP, which is an Arg in the PKS enzyme. The functional role of this residue was described as a “lid” that closes over aromatic substrates upon binding as determined by X-ray crystallography for the benzhydroxamic acid complex of HRP-C [25]. The replacement of the aromatic phenyl group with the positively charged guanido group in arginine would be expected to significantly alter substrate-binding kinetics and equilibrium.

### 3.3. Kinetic Characterization of PKS

**Figure 4:**
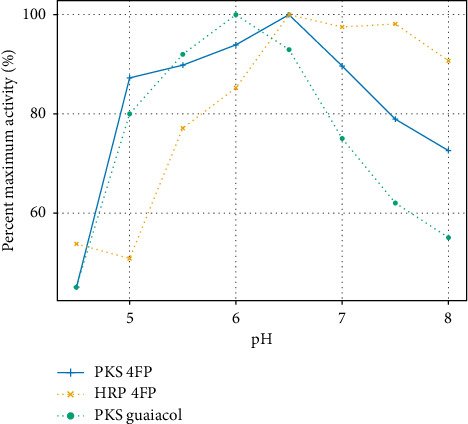
Effect of pH on activity of the PKS enzyme in the oxidation of 4FP and guaiacol. For comparison, the pH dependence of HRP in the 4FP reaction is also shown.

Optimum pH for the PKS enzyme was determined using both the guaiacol oxidation and 4FP oxidation reactions. The data presented in Figure 4 show the relative activities of the enzyme in both assays as a function of pH, where activity is presented as a percentage of the maximum activity observed. For the PKS enzyme, the optimal pH was found to be between 6.0 and 6.5 for both the assays used (4FP and guaiacol oxidation), though greater than 80% of the maximal activity was maintained as low as pH 5. This is in contrast to the HRP catalyzed oxidation of guaiacol, where at pH 5.0, the reaction occurred at only 50% of its maximum. On the other hand, PKS activity dropped to between 55 and 72% at pH 8, while HRP retained 90% of its activity. The slight difference in the activity of PKS in the guaiacol vs. 4FP reactions observed at higher pH may be related to the more complex stoichiometry of the guaiacol reaction compared with 4FP. Alternatively, slight differences in the pKa of the phenolic substrates could also account for the observed differences. Overall, the data indicate that PKS may be a better low pH catalyst, while HRP maybe better for higher pH applications.

**Figure 5:**
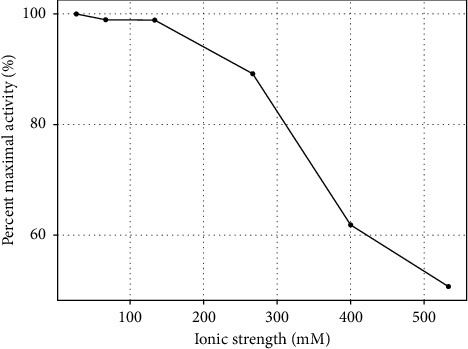
Effect of ionic strength on activity of PKS in the oxidation of 4FP to benzoquinone.

**Figure 6:**
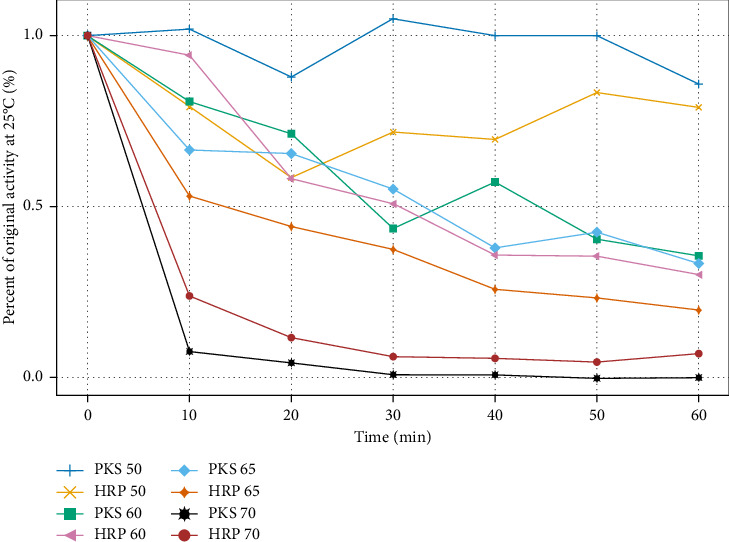
Evaluation of the thermal stability of the PKS and HRP enzymes. Each enzyme was preincubated at the corresponding temperatures for the designated time and immediately assayed using 4FP oxidation as a probe for activity.

Using buffers at the optimal pH of 6 for PKS, the effect of ionic strength was examined by carrying out the 4FP oxidation in buffers ranging from 20 mM to 400 mM potassium phosphate. The data for these experiments are shown in Figure 5, which demonstrates a threshold value for ionic strength of about 200 mM. Above this concentration, activity decreased steadily and started to plateau around 50% activity. Whether this is an ionic strength effect, or an effect of high concentrations of phosphate on the enzyme is unclear, as Bovaird et al. have shown an irreversible inactivation of HRP in 100-mM phosphate buffer, pH 5.0 [26]. To address this, addition of increasing concentrations of NaCl up to 400 mM was made to the 50-mM phosphate buffer at pH 6.0, and a similar inhibitory effect on the activity was observed as with the 400 mM phosphate alone, suggesting the decrease in activity is primarily ionic strength-dependent. An additional interesting feature (not shown in Figure 5) was that the addition of 400-mM NaCl to the 400-mM phosphate reaction did not result in further reduction of activity, rather the activity vs. ionic strength curve continued to plateau at around 50% reduction. The molecular basis for this is not immediately apparent but could involve enhanced substrate binding via hydrophobic interactions at higher ionic strength possibly compensating for reduced catalytic activity, since it is well known that hydrophobic interactions can be strengthened at high ionic strength. From the pH and buffer concentration studies, an optimal condition of 50-mM phosphate buffer at pH 6.0 was selected for additional experiments to assess temperature stability on the reaction with 4FP. Enzyme samples were prepared in optimized buffer and preincubated for 0–60 min at temperatures ranging from 50–70°C. As indicated in Figure 6, both HRP and PKS remain stable for up to 60 min at 50°C; however, above this temperature, both of them lost activity during the preincubation steps. These data show that the maximum sustained temperature at which PKS exhibits stability between 50 and 60°C, consistent with other plant peroxidases studied [27] including HRP, as determined in this study.

### 3.4. Michaelis–Menten Analysis

Michalis–Menten kinetic analysis of the PKS, using both guaiacol and 4FP substrates, was carried out in 50-mM phosphate buffer pH 6.0. With both substrates, saturation kinetics was observed with increasing substrate concentration. The rate of 4FP oxidation was measured by monitoring the quinone product formation by HPLC using the optimum absorbance of 254 nm for the quinone, while guaiacol oxidation was monitored spectrophotemetrically by detection of the tetraguaiacol product at 470 nm over 1 min. The *k*_cat_ and *K*_*m*_ values determined with each substrate are given in Table 1. For both substrates, a *K*_*m*_ of 3.0 mM was observed; however, the turnover number observed for the degradation of guaiacol was substantially higher than that of 4FP (105,000 min^−1^ vs. 68,000 min^−1^), which is most likely due to the higher reduction potential of the fluorinated substrate.

### 3.5. Spectrophotometric Properties of PKS

**Figure 7:**
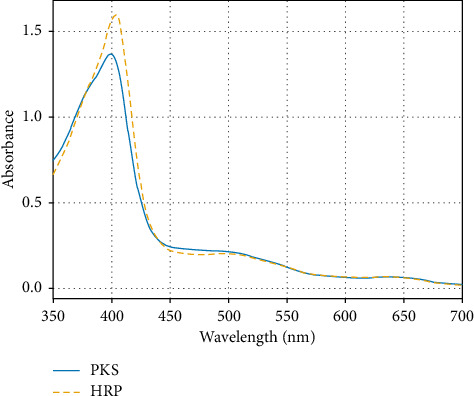
Absorption spectra for PKS and HRP in 50 mM phosphate buffer pH 7.0. Both enzymes were at a concentration of 16 μM.

**Figure 8:**
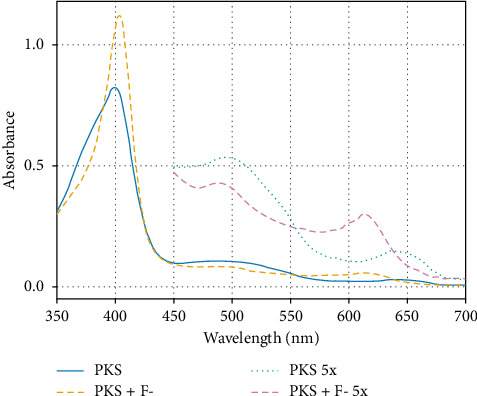
Absorption spectra of the native PKS enzyme in 50 mM potassium phosphate buffer at pH 7.0 in the absence and presence of 50 mM KF. The region between 450–700 nm was magnified 5x to show the precise wavelengths for the CT bands. Concentration of the enzyme was 10 μM.

The initial characterization of the PKS enzyme involved electronic absorption analysis in 50-mM pH 7.0 potassium phosphate buffer and comparison with HRP.  Figure 7 shows the absorption spectra of the two enzymes under identical conditions. Both display prominent Soret peaks along with nearly identical β-bands and porphyrin-to-metal charge transfer bands at 500 nm and 643 nm, respectively. For the HRP, a characteristic Soret with a maximum absorbance at 403 nm was observed and a prominent shoulder at 386 nm, which is consistent with a mixture of 5-coordinate and 6-coordinate HS ferric species similar to that previously reported for the HRPC enzyme [28, 29]. Interestingly, in the PKS spectrum, the Soret maximum is slightly blue-shifted by about 4 nm–399 nm, with a slightly blue-shifted shoulder at 382 nm. The extinction coefficient for the PKS enzyme was determined by measuring the total heme content of the purified enzyme sample by HPLC and using this as the concentration of the purified enzyme. This method has been validated using HRP as a standard heme protein. An ε_399_ value of 8.5 × 10^4^ M^−1^ was determined for the native PKS enzyme at pH 7.0. It should be noted here that minor perturbations in the absorption spectra occur with changing pH, so quantitation of enzyme using this extinction coefficient was always carried out at pH 7.0. Addition of potassium fluoride (200 mM) to the enzyme resulted a shift in the Soret to a sharp peak to 407 nm, as shown in Figure 8, with increase in molar absorptivity consistent with conversion of the iron to the HS 6c ferric complex in which the fluoride ion directly coordinates to the iron, as observed in HRP [30, 31]. An ε_407_ value of 1.1 × 10^5^ M^−1^ was determined for the 6c-HS fluoride complex for PKS. The weak-field ligand, fluorine, caused a substantial blue shift in the CT region from 643 nm to 620 nm and from 500 nm to 490 nm, also as observed in HRP [32]. The implications for this in defluorination reactions is potentially significant, particularly in light of the recent studies highlighting PKS as an active catalyst in degradation of fluorinated phenols [16], presumably via a mechanism in which the fluorine atoms are removed as F^−^ ions [33]. Inhibition of peroxidases by F^−^ has been documented [34], although not specifically with HRP.

**Figure 9:**
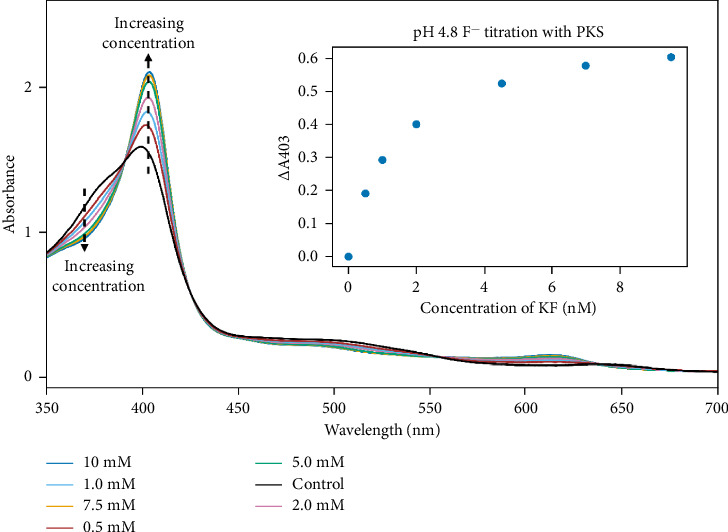
Spectral changes during the titration of PKS (18 μM) with KF in 50 mM phosphate buffer at pH 4.8. Inset shows binding curve from which the dissociation constant was calculated.

The PKS enzyme was initially titrated with KF at a pH of 4.8 in order to determine the dissociation constant for F^−^ where the absorbance values at 407 nm vs. KF concentration were used to calculate *K*_*d*_, as shown in Figure 9. A typical saturation curve was observed over a range of KF concentrations between 1.0 and 10.0 mM, and graphical analysis of the data yielded a *K*_*d*_ value of 1.3 mM for the F^−^ under these conditions. It should be noted that a strong pH-dependence for fluoride binding to various heme proteins has been reported, with lower pH yielding much tighter binding, indicating HF is the preferred binding species [35]. For example, for HRP, *K*_*d*_ values ranging from 0.60 mM at pH 4.0 up to over 1.0 M at pH 8.0 were observed [36]. Therefore, the *K*_*d*_ value for F^−^ binding to PKS was determined at an additional pH value (6.0) for comparison (data not shown). Binding of F^−^ to PKS at higher pH (6.0) was weaker than that at pH 4.8 as indicated by the larger *K*_*d*_ value of 9.4 mM compared to the 1.3-mM *K*_*d*_ measured at pH 4.8. The conserved Arg-38 and His-42 in ligand binding at the active site of HRP have been implicated in this behavior, thus the similar observations made in F^−^ binding to PKS is not surprising, as these are also retained in PKS [37–39]..

### 3.6. Trifluoroacetate Binding to PKS

**Figure 10:**
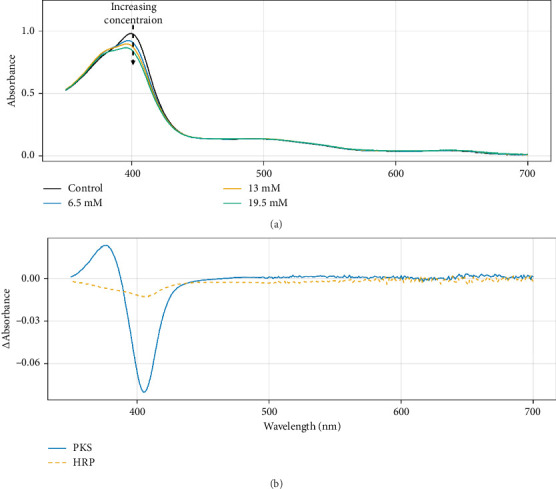
(a) Spectral changes of the PKS enzyme upon titration with trifluoroacetic acid in 50-mM potassium phosphate buffer, pH 5.0. (b) Comparison of difference spectra for the binding of TFA to PKS and HRP at 6.5-mM TFA at pH 5.0.

Carlsson et al. demonstrated that in HRP, acetate binding to the active site heme showed distinct spectral features compared with fluoride binding [40]. Specifically, as opposed to forming a red-shifted Soret peak at 407 nm, as with the weak-field ligand fluoride, the difference spectrum for the acetate complex vs. native HRP had a slight increase at 386 with a decrease at 406. The authors use EPR and X-ray crystallography to develop a binding model in which acetate stacks parallel to the heme plane and forms strong hydrogen bonds with an active site Arg, preventing the binding of solvent to the axial position on the heme. Although both HRP and PKS demonstrated similar effects with acetate in the current study (data not shown), the perfluorinated derivative, trifluoroacetate, only formed the analogous complex with the PKS enzyme, and not HRP.  Figure 10(a) shows changes in the absorption spectra of PKS upon addition of trifluoroacetate, demonstrating clear binding of this ligand. Since TFA is a stronger acid than acetate, the pH of the reaction was monitored over the course of the titration and although slight changes in pH were observed, control spectral data were acquired where HCl was added to simulate these minor changes, and no spectral changes were observed, confirming the changes were due to TFA binding, and not pH changes in the solution.  Figure 10(b) shows the corresponding difference spectra for the titration of both PKS and HRP with TFA. Qualitatively, the difference spectrum for the PKS enzyme is the same as that observed with acetate, while the HRP showed no spectral changes. The data suggest that TFA binds in a similar parallel fashion to the heme cofactor in PKS. It is interesting that under the same conditions, TFA did not appear to form the same complex with HRP. With concentrations up to 30-mM TFA, no spectral shift consistent with the displacement of an axial ligand was observed, in fact the slightest shift toward the 6c complex was detected based on changes in the CT region. The crystal structure for the acetate complex of HRP has been reported [40] and shows the methyl group of the acetate molecule tightly packed into the distal pocket of the heme site with hydrophobic contacts with Pro-139, Phe-41, and Phe-45, all of which are conserved in the PKS sequence. As mentioned previously, however, there is a Phe ⟶ Arg substitution in PKS at Position 68 where in HRP, the Phe is thought to protect the access channel. This modification may provide greater access to more hydrophilic substrates resulting in higher “on” rates for binding, thus altering the equilibrium binding, and explaining the observed behavior with TFA.

**Figure 11:**
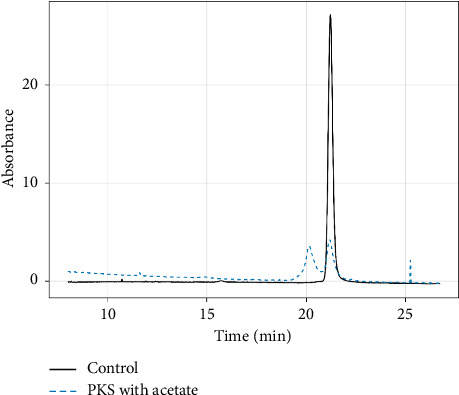
HPLC chromatogram showing unmodified heme in PKS and following 1 hr incubation with H_2_O_2_ in 300-mM acetate buffer. The adduct was monitored at a wavelength of 400 nm.

Huang et al. have also examined acetate binding to HRP along with the subsequent oxidation of the bound acetate via the catalytic cycle of HRP, resulting in covalent modification of the prosthetic heme group and consequent enzyme inactivation [22]. The reaction occurs via radical initiated formation of an isoporphyrin intermediate, as described previously with HRP reaction with alkyl hydrazines [41] as well as in the inactivation of CYP450_BM3_ (F87G) with aromatic aldehydes [42]. That PKS also capable of this carboxylic acid oxidation was demonstrated using protocols established in this study. PKS was incubated in the presence of concentrations of acetate buffer ranging from 50 to 400 mM along with 1.0 mM hydrogen peroxide at several different pH values. As with HRP, PKS showed optimal heme modification around pH 4.8, and at the highest concentration of acetate (Figure 11). At lower pH, only heme destruction was observed with no stable heme adducts, and at higher pH, little to no heme modification/degradation was observed. Given the analogous spectral changes observed in PKS using TFA, this PFOA was also examined for evidence that PKS could oxidize it via a similar mechanism. As with acetate, significant heme degradation was observed in the presence of TFA at concentrations above 50 mM. However, no detectable heme adducts were observed in this reaction. Similar observations were made using HRP enzyme as a control. One important distinction between the acetate and trifluoroacetate is the pKa value of the acid form, with acetic acid having a pKa of 4.76, while that of TFA is 0.23. Consequently, the anionic form of TFA would be the dominant form at any pH used. Since prior studies suggested that the protonated form of acetic acid was required for adduct formation, it is not surprising then that TFA did not yield any such adduct; however, the rapid degradation of the heme in the presence of TFA suggest that some type of chemical activation of the fluorinated carboxylic acid did occur.

## 4. Conclusion

In summary, we have demonstrated a simple and efficient method of purification for the novel peroxidase catalyst from PKS and identified optimal pH and ionic strength conditions for catalysis. Spectral characterization of the enzymes reveals clear similarities in its properties to those of the well-characterized Class III peroxidase from horseradish root with several notable differences. These differences include the following: (1) dissimilarities in binding affinity toward F^−^ ions, (2) slight blue shift and greater asymmetry in the Soret peak, with lower molar absorptivity, and (3) observable shift in the Soret peak upon binding trifluoroacetic acid. Differences in binding of TFA may be related to specific structural differences in the binding site of the PKS enzyme vs. HRP as determined by the primary and tertiary structure determined for PKS in this study. Overall, this work lays the foundation for the development of this highly active, highly abundant peroxidase for future biotechnological applications, including potential use in degradation of fluorinated organics of different classes. For instance, the strategic integration of these low-cost PKS peroxidase enzymes with photocatalytically enhanced high-surface-area substrates, as described in prior studies [43–45], could enable the development of economically viable bioreactors exhibiting high efficacy in the degradation of per- and polyfluoroalkyl substances, and perhaps other halogenated organic pollutants.

## Figures and Tables

**Table 1:** The Michaelis–Menten data for the degradation with PKS.

Substrate	*K*_*m*_ (mm)	*k*_cat_
4-Fluorophenol	3.0	1100 ± 54
Guaiacol	3.0	1730 ± 42

## Data Availability

The data that support the findings of this study are available from the corresponding authors upon reasonable request.
